# Editorial: Enteric Unicellular Eukaryotic Parasites and Gut Microbiota: Mechanisms and Ecology

**DOI:** 10.3389/fmicb.2021.779412

**Published:** 2021-10-25

**Authors:** Magali Chabé, Gabriela Certad, Simone Mario Caccio

**Affiliations:** ^1^University Lille, CNRS, Inserm, CHU Lille, Institut Pasteur de Lille, U1019—UMR 9017—CIIL—Centre d'Infection et d'Immunité de Lille, Lille, France; ^2^Délégation à la Recherche Clinique et à l'Innovation, Groupement des Hôpitaux de l'Institut Catholique de Lille, Lille, France; ^3^Department of Infectious Diseases, Istituto Superiore di Sanità, Rome, Italy

**Keywords:** intestinal protozoa, gut microbiota, parasite detection, metagenomics, eukaryome

Human-associated gut microbial communities, which play a central role in human health and disease, are composed not only of bacteria, but also of viruses, archaea and eukaryotes (fungi, helminths, and protozoa). A growing literature is starting to reveal how gut eukaryotic parasites and the gut bacterial microbiota may interact in various vertebrate hosts (Leung et al., 2018). For example, it has been shown that bacterial and eukaryotic microorganisms residing in the human gut can affect each other's pathogenicity (Leung et al., 2018). Among these eukaryotes, protozoa are currently the subject of studies addressing their effects on the gut bacterial microbiota. To date, most studies have focused on well-known pathogenic protozoa such as *Giardia, Cryptosporidium*, and *Entamoeba histolytica*. In parallel, we know very little about the ecological interactions between gut bacteria and intestinal protozoa that are either non-pathogenic or whose pathogenicity is unknown or controversial. Some of these protozoa might even be beneficial (Lukeš et al., 2015); for example, *Blastocystis*, whose pathogenicity remains controversial, could be considered as a member of the healthy human gut community (Chabé et al., 2017).

Despite recent and significant advances, many questions remain unanswered regarding the effects of intestinal protozoa on the gut bacterial microbiota or *vice versa*. Therefore, the main objectives of this special issue were to cover methodological aspects (i.e., tools available and required for the detection of these unicellular parasites), and to present relevant examples of the complex interaction between protozoa and bacteria. In fact, their detection is not always a straightforward issue in complex matrices such as feces, but is the prerequisite for an accurate study of the interaction between intestinal protozoa (considered pathogenic or potentially beneficial) and the gut microbiota.

The Research Topic belongs to the Microbial Symbioses section and comprises 5 articles: one review, two perspectives, and two original research papers. As Topic Editors, we provide a brief overview of these contributions, starting with the two perspective papers that deal with methodological aspects (Franssen et al.; Wylezich and Höper). In the first contribution, Franssen et al. highlighted how metagenomics datasets, generated using untargeted (shotgun) sequencing of nucleic acids extracted from environmental samples, can be interrogated to detect specific eukaryotic sequences. The authors provided a proof of principle using *Cryptosporidium* as a test organism, and compared various bioinformatics approaches in terms of sensitivity and specificity of detection, while underlining the need for further optimization. In the second contribution, Wylezich and Höper proposed to better exploit rRNA sequence-based metagenome datasets to detect parasites from various matrices such as tissues or feces. This method, that the authors referred to as “Meta-Ribosomalomics,” allows the simultaneous investigation of prokaryotic and eukaryotic diversity in the same sample. Overall, these kinds of diagnostic metagenomics provide an unbiased and holistic view of the eukaryotic diversity existing in the intestinal tract.

Mammeri et al. studied for the first time the impact of *Cryptosporidium parvum* infection on the gut microbiome of goat kids, an important natural host of this parasite. Since studies on *Cryptosporidium*-microbiota interactions are scarce, the work of Mammeri et al. reported new and interesting insights into the modulation of taxonomic and functional profiles of the gut microbiome induced by cryptosporidiosis. They described a dramatically decrease of butyrate-producing bacteria, that could explain the intestinal inflammation associated with *C. parvum* infection.

In a comprehensive review, Fekete et al. summarized the current knowledge of the effect of *Giardia* on the gut microbiota or *vice versa*. The authors showed the complexity of these interactions, and pointed to the central role played by bacteria in determining susceptibility or resistance to colonization by *Giardia*. In turn, *Giardia* induces functional and compositional alterations to the microbiota, which lead to systemic effects for the host that potentially persist even after parasite clearance. In polymicrobial infections, co-infections with *Giardia* can be deleterious for the host (i.e., *Giardia* and enteroaggregative *Escherichia coli* co-infection decreases immune functions enhancing growth impairment in malnourished children). In turn, co-infections with this protozoan can also be associated with a protective effect against enteropathogen-driven diarrhea due to the immunomodulatory properties of *Giardia*.

Beside *Cryptosporidium* and *Giardia*, two well-known intestinal pathogens, *Blastocystis* seems to act rather as a gut commensal protozoan. The research paper of Billy et al. focused on the effect of *Blastocystis* subtype ST3 colonization on the clinical outcome and gut microbiota composition in an experimental rat model of chemically induced colitis. Interestingly, Billy et al. found that short-term (i.e., 3 weeks) colonization of *Blastocystis* ST3 had no effect on the manifestation of colitis in the rat model, while long-term (i.e., 13 weeks) *Blastocystis* colonization seemed to promote a faster recovery from colitis. Moreover, a higher bacterial diversity, considered a hallmark of a healthy gut, was recorded 2 days after colitis induction in rats after long-term *Blastocystis* ST3 colonization.

Finally, this collection, by providing a brief overview of the various interactions between intestinal protozoa and gut bacterial microbiota, paves the way to further studies on this topic. Although bacteria represent the most abundant and diverse biome in our gut, we argue that interactions with eukaryotes play an important role in gut health and disease. These interactions are currently not fully understood, and clearly deserve to be further explored.

## Author Contributions

All authors listed have made a substantial, direct and intellectual contribution to the work, and approved it for publication.

## Conflict of Interest

The authors declare that the research was conducted in the absence of any commercial or financial relationships that could be construed as a potential conflict of interest.

## Publisher's Note

All claims expressed in this article are solely those of the authors and do not necessarily represent those of their affiliated organizations, or those of the publisher, the editors and the reviewers. Any product that may be evaluated in this article, or claim that may be made by its manufacturer, is not guaranteed or endorsed by the publisher.
